# Two-stage sparse multi-objective evolutionary algorithm for channel selection optimization in BCIs

**DOI:** 10.3389/fnhum.2024.1400077

**Published:** 2024-05-22

**Authors:** Tianyu Liu, Yu Wu, An Ye, Lei Cao, Yongnian Cao

**Affiliations:** ^1^School of Information Engineering, Shanghai Maritime University, Shanghai, China; ^2^Tiktok Incorporation, San Jose, CA, United States

**Keywords:** multi-objective evolutionary algorithm, channel selection, two-stage framework, sparse initialization, score assignment strategy

## Abstract

**Background:**

Channel selection has become the pivotal issue affecting the widespread application of non-invasive brain-computer interface systems in the real world. However, constructing suitable multi-objective problem models alongside effective search strategies stands out as a critical factor that impacts the performance of multi-objective channel selection algorithms. This paper presents a two-stage sparse multi-objective evolutionary algorithm (TS-MOEA) to address channel selection problems in brain-computer interface systems.

**Methods:**

In TS-MOEA, a two-stage framework, which consists of the early and late stages, is adopted to prevent the algorithm from stagnating. Furthermore, The two stages concentrate on different multi-objective problem models, thereby balancing convergence and population diversity in TS-MOEA. Inspired by the sparsity of the correlation matrix of channels, a sparse initialization operator, which uses a domain-knowledge-based score assignment strategy for decision variables, is introduced to generate the initial population. Moreover, a *Score*-based mutation operator is utilized to enhance the search efficiency of TS-MOEA.

**Results:**

The performance of TS-MOEA and five other state-of-the-art multi-objective algorithms has been evaluated using a 62-channel EEG-based brain-computer interface system for fatigue detection tasks, and the results demonstrated the effectiveness of TS-MOEA.

**Conclusion:**

The proposed two-stage framework can help TS-MOEA escape stagnation and facilitate a balance between diversity and convergence. Integrating the sparsity of the correlation matrix of channels and the problem-domain knowledge can effectively reduce the computational complexity of TS-MOEA while enhancing its optimization efficiency.

## 1 Introduction

Brain-computer interface systems (BCIs) have garnered increasing attention within academic and industrial circles due to their broad real-world applications. By acquiring brain signals, BCIs facilitate external device control and communication without necessitating physical movement. For instance, individuals with paralysis can employ BCIs to manage external devices like wheelchairs, prosthetics, and robots, consequently enhancing their quality of life (Krishna Rao et al., 2022). Furthermore, BCIs find utility in monitoring patients' cerebral functions within the medical domain and delivering enhanced immersive experiences in the realm of gaming (Qu et al., 2023).

The majority of existing non-invasive BCIs utilize external sensors equipped with multiple channels (32 channels, 62 channels, or even more) for the acquisition of electroencephalography (EEG) signals (Sibilano et al., 2024). More channels lead to a more comprehensive capture of EEG signals. However, owing to the impact of the skull and scalp on electrical signal transmission, EEG signals obtained through external sensors frequently contain noise and extraneous information irrelevant to specific tasks. Moreover, the extensive number of EEG channels compounds the difficulties in data collection and significantly increases the computational complexity of processing this data, leading to increased and often unnecessary computational costs. Therefore, selecting appropriate channels (known as channel selection optimization) from the entirety has emerged as a pivotal challenge in the realm of BCIs (Almanza-Conejo et al., 2023).

A significant portion of research in channel selection optimization is grounded in EEG signal analysis (Martínez-Cagigal et al., 2022). The channel selection method based on signal analysis begins by extracting and selecting features from EEG signals, subsequently choosing the most suitable subset of channels for a specific task. On the one hand, such methods require users to have domain-specific expertise related to the task; otherwise, it may lead to selecting sub-optimal channel subsets. Furthermore, the pre-designed algorithmic workflow may become entirely inappropriate if the task changes. On the other hand, most signal analysis-based channel selection methods focus on a single optimization objective, with task accuracy often chosen as the optimization goal in many algorithms (Rocha-Herrera et al., 2022). However, in addition to task accuracy, the number of selected channels is also a crucial metric when conducting channel selection. This is because the number of channels adopted will determine the convenience of using BCI devices. However, there is typically a trade-off between the number of selected electrodes and task accuracy. Therefore, an efficient channel selection approach must strike a compromise between the number of selected channels and task accuracy since these two factors are mutually exclusive. In this context, the utilization of multi-objective evolutionary algorithms (MOEAs) (He et al., 2022), recognized for their efficiency in resolving problems with conflicting multiple objectives, has captured the attention of researchers. In most multi-objective channel selection algorithms, the number of selected channels (or the number of deleted channels) and the accuracy of tasks are directly employed to construct the multi-objective problem model (Alotaiby et al., 2015). However, researchers have found that utilizing the aforementioned multi-objective problem model can sometimes lead to premature convergence of MOEAs (Abdullah et al., 2022). Consequently, developing a well-designed and practical multi-objective problem model becomes a critical factor influencing the performance of multi-objective channel selection algorithms.

**Figure 1:**
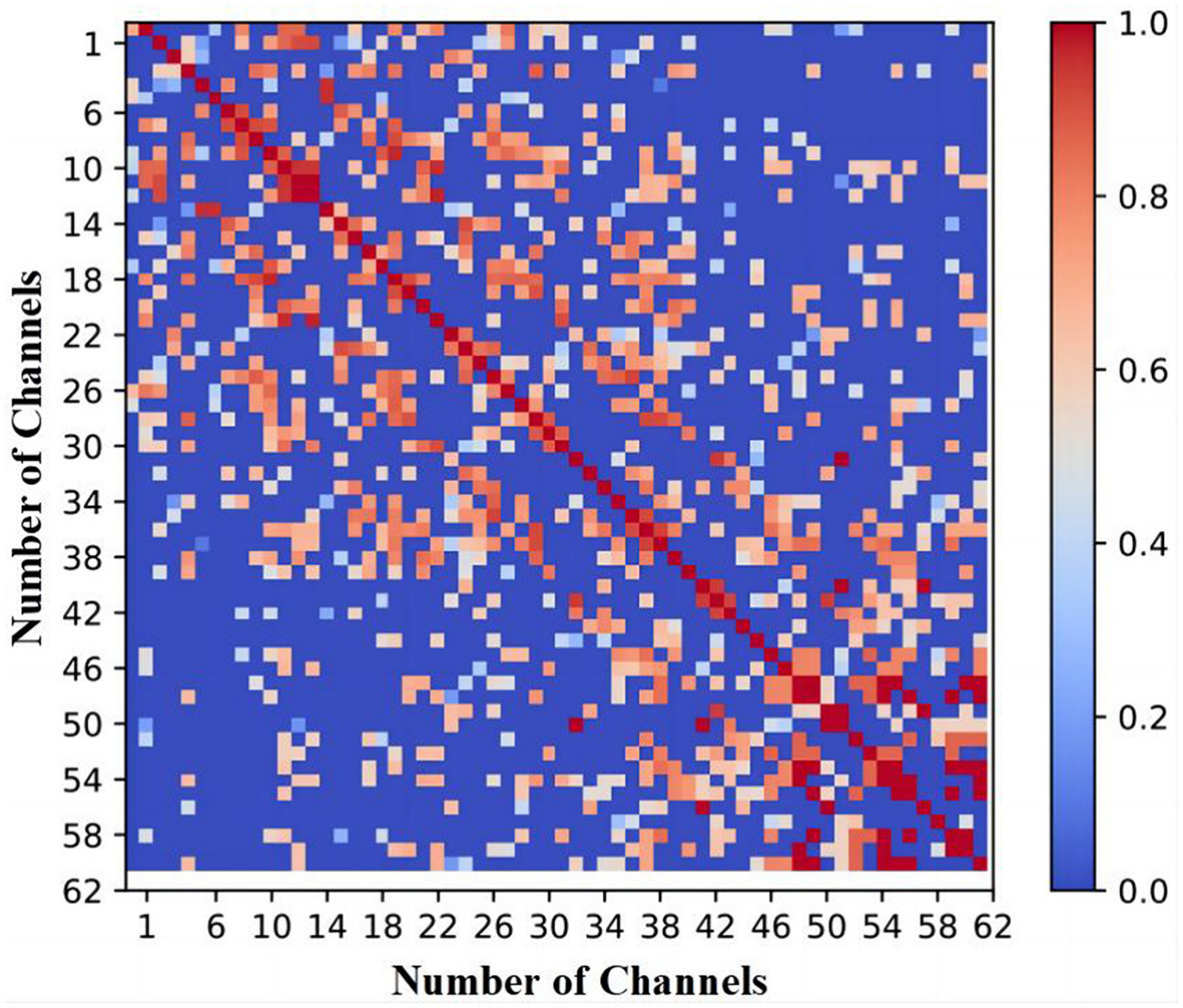
Correlation matrix of 62 channels under 94% accuracy for a fatigue detection task.

Studies have demonstrated that connectivity information can effectively capture the attributes of EEG signals, given that interactions and collaborations among various regions shape the neural activity in the brain (Moon et al., 2020). Recently, there has been a rising trend in utilizing the correlation matrix of channels to address channel selection optimization problems. This is primarily because the correlation matrix can depict the interactions and cooperative activities among different brain regions (Liu and Ye, 2023). It has been demonstrated that due to the non-uniform connectivity patterns in the brain, the correlation matrix of EEG signals is typically sparse (Liu et al., 2019). For example, Figure 1 exemplifies the correlation matrix of 62 EEG channels utilized in a fatigue detection task with a classification accuracy of 94%. In Figure 1, the red color represents that the corresponding two channels are entirely linearly correlated (linear correlation coefficient of 1), while the blue color indicates that the two channels are linearly independent (linear correlation coefficient of 0). It can be observed from Figure 1 that the majority of cells in the correlation matrix are depicted in blue, indicating that the correlation coefficients of the corresponding elements are close to zero, thereby demonstrating the sparse nature of the correlation matrix. However, few studies consider the sparsity of the correlation matrix when adopting it for solving channel selection problems.

This paper introduced a two-stage sparse multi-objective evolutionary algorithm (TS-MOEA), tailored for optimizing channel selection in BCIs. To prevent the algorithm from stagnating, TS-MOEA employs a two-stage framework. In this framework, the entire optimization process is divided into two phases, namely the early and late stages, with each stage addressing different multi-objective problem models. Specifically, in the early-stage phase, the adopted objective function is more sensitive to the deletion of channels to prevent the algorithm from falling into local optima. Furthermore, inspired by the sparsity of the correlation matrix of channels, a sparse initialization operator is employed when initializing the population in TS-MOEA. In the sparse initialization operator, a domain knowledge based strategy, which utilizes channels' positions and distance matrix, is used to assign scores to decision variables. Additionally, in the early stage of the algorithm, a *Score*-based mutation strategy is employed to enhance the search efficiency of the algorithm. In summary, the algorithm presented in this paper differs from existing multi-objective lead optimization algorithms in three main aspects. First, the proposed algorithm employs two distinct multi-objective optimization models, whereas current methods optimize for a single multi-objective model throughout the entire search process. Second, the proposed algorithm analyzes the sparsity of the channel correlation matrix and incorporates a sparsity-based strategy in the design of the operators to enhance the efficiency of the algorithm. Lastly, the proposed algorithm utilizes domain-specific knowledge to guide the search process of the algorithm. The primary contributions of this paper are outlined as follows:

1. A two-stage framework is employed in this study to assist the algorithm in escaping local optima. This framework divides the optimization process into the early and late stages, and different multi-objective problem models are used in the two stages.
2. Inspired by the sparsity observed in the correlation matrix of channels, a sparse initialization operator is introduced to create the initial population. Within this operator, a strategy based on domain knowledge is employed, leveraging channels' positions and distance matrix to allocate scores to decision variables.
3. A *Score*-based mutation strategy is employed to enhance the search efficiency in the early stage of TS-MOEA.
4. The performance of TS-MOEA and five other advanced multi-objective algorithms has been evaluated using a 62-channel EEG-based BCI system for a fatigue detection task.

The remainder of this paper is structured as follows: Section 2 presents the relevant background theory, followed by the description of the proposed TS-MOEA in Section 3. Section 4 covers the experiment and result analysis. Section 5 contains a discussion of the parameters and results, while Section 6 presents the concluding remarks.

## 2 Backgrounds

### 2.1 Acquisition and processing of EEG signals

**Figure 2:**
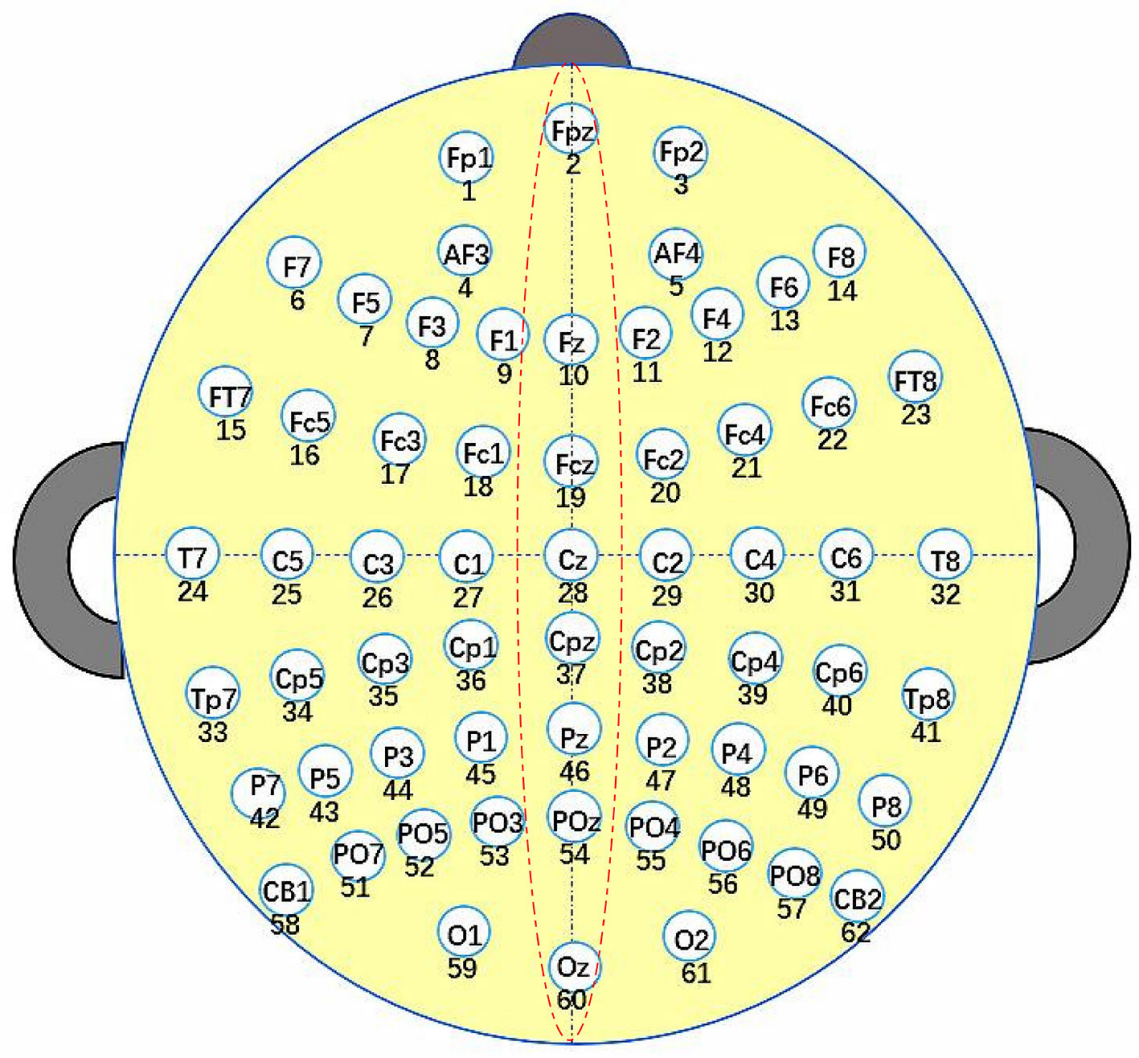
Electrode placement follows the standard International 10–20 System.

In this study, EEG signals were collected using an ESI-64 high-resolution system (SynAmps2, Neuroscan) with 62 EEG channels (Chen et al., 2022). These 62 electrodes were positioned in accordance with the international 10–20 standard, as depicted in Figure 2. The initial sampling frequency was 1,000 Hz, which was down-sampled to 250Hz for data processing. Subsequently, the recorded signals underwent filtering with the frequency from 0 to 40 Hz. The raw EEG signals were sampled every 5 seconds, undergoing conversion from analog to digital signals through the utilization of a sampling window and a sliding window of 5 seconds. In this paper, the bilateral linked mastoid (LM) (Scannella et al., 2016), which is the average of the left and right mastoids, was used as the reference signal during the acquisition of EEG signals.

This study utilizes the correlation matrix to describe the characteristics of the collected EEG signals. In recent times, the Pearson Correlation Coefficient (PCC) (Pearson, 1895), along with the Phase Locking Value (PLV) (Aydore et al., 2013), and Transfer Entropy (TE) (Schreiber, 2000), have become extensively used methods for calculating the correlation coefficient between EEG signals in BCIs.

PCC quantifies the linear correlation between two signals, with its value ranging from -1 to 1. A PCC value of 0 signifies that the signals are linearly uncorrelated. Conversely, PCC values of -1 and 1 indicate negative and positive linear relationships, respectively. Consider $X_{i}=\left\{x_{i}^{1},x_{i}^{2},...,x_{i}^{T}\right\}$ as the EEG signal from the *i*^*th*^ channel, where *T* represents the signal's length, and μ_*i*_ and σ_*i*_ are the mean and standard deviation of the *i*^*th*^ signal. The PCC value between *X*_*i*_ and *X*_*k*_ is computed using Equation (1).


(1) $$PCC(i,k)=\frac{\frac{1}{T}\sum_{t=1}^{T}\left(X_{i}^{t}-\mu_{i})(X_{k}^{t}-\mu_{k}\right)}{\sigma_{i}\sigma_{k}}$$

PLV is used to describe phase synchronization between two signals by averaging the absolute phase differences. PLV can be calculated using Equation (2). In this equation, $\varphi_{i}^{t}$ denotes the phase of the signal at time point *t* for the *i*^*th*^ signal.


(2) $$PLV(i,k)=\frac{1}{T}\left|\sum_{t=1}^{T}e^{j(\varphi_{i}^{t}-\varphi_{k}^{t})}\right|$$

TE is a metric for quantifying the directed information flow from signal *X*_*i*_ to *X*_*k*_, as delineated in Equation (3). Essentially, TE assesses the extent to which signal *X*_*i*_ can improve the prediction of signal *X*_*k*_. A TE value of 0 indicates the absence of a causal relationship between the two time series, implying that knowing the past values of *X*_*i*_ may not aid in predicting *X*_*k*_.


(3) $$TE(i\to k)=\frac{1}{T-1}\sum_{t=1}^{T-1}p(X_{i}^{t},X_{k}^{t},X_{k}^{t+1})\log\frac{p(X_{k}^{t+1}|X_{k}^{t},X_{k}^{t})}{p(X_{k}^{t+1}|X_{k}^{t})}$$

Researches have shown that the performance of TE is relatively worse than that of PCC and PLV. PLV performs better than PCC slightly (Gong et al., 2024). However, PCC is faster in terms of computational speed because of its simplicity (Maria et al., 2023). In this case, this paper utilizes PCC to obtain the correlation matrix of EEG signals in terms of both performance and computational speed.

### 2.2 MOEAs

Multi-objective optimization problems (MOPs) are prevalent in various real-world scenarios, characterized by multiple conflicting objectives. The general formulation of a maximum MOP is represented in Equation (4), where **x** = (*x*_1_, ..., *x*_*n*_)∈Ω denotes the solution *x* within a search space of dimension *n*, and Ω represents the feasible region in the search space. *M* corresponds to the number of objectives considered in the optimization problem. For an MOP with multiple exclusive objectives, the optimization algorithms can not find a single optimal solution that simultaneously optimizes all objectives. This is due to the exclusive between objectives, where enhancing the performance of one objective may lead to a decline in others. Consequently, the goal of MOEAs is to identify a set of Pareto optimal solutions. x^*^ is regarded as a Pareto optimal solution if there is no other solutions that can dominate x^*^. Suppose a maximum MOP as shown in Equation (4), *x* dominates x^*^ if and only if $\forall i\in \left\{1,2,...,M\right\},f_{i}\left(\text{x}\right)\geq f_{i}\left(x^{*}\right)$ and $\exists i\in \left\{1,2,...,M\right\},f_{i}\left(\text{x}\right)>f_{i}\left(x^{*}\right)$. Being population-based search methods, evolutionary algorithms (EAs) have proven to be efficient tools for tackling MOPs by generating a collection of candidate solutions within a single execution.


(4) $$\begin{array}{l} Maximum\;F\left(X\right)=\left(f_{1}\left(X\right),f_{2}\left(X\right),...,f_{M}\left(X\right)\right) \end{array}$$

Current MOEAs can be categorized into three main types: dominance-based, decomposition-based, and index-based algorithms. For MOEAs belonging to the first category, the basic idea is to determine the priority of one solution by the dominant relation between the solution and the others. The typical algorithm in the first category is NSGA-II (Deb et al., 2002). In NSGA-II, a fast non-dominated sort method, which are widely adopted in dominance-based MOEAs, is proposed. Many improved algorithms have been submitted in recent years. For example, in CBGA-ES+ (Pradhan et al., 2021) proposes a hybrid selection strategy combining cluster-based methods and the traditional non-dominated elitist selection method to select parent solutions. In Premkumar et al. (2021), a MOSMA, which combines the Slime Mould Algorithm and the traditional NSGA-II, is proposed to solve MOPs in industries. In the CMMO (Ming et al., 2023) algorithm, a cooperative evolution strategy, combined with customized environmental and mating selection, forms the basis for addressing MOPs. The algorithm utilizes dynamically adjusted relaxation factors to retain advantageous solutions with diverse decision spaces. This algorithm exhibits outstanding performance in solving multi-modal multi-objective problems. In ASDNSGA-II (Deng et al., 2022), a special congestion degree strategy and a new adaptive crossover operator are proposed to improve the performance of NSGA-II when handling multi-modal MOPs.

For MOEAs based on decomposition, the basic idea is to translate the original MOP into a set of single-objective problems [as seen approaches like MOEA/D (Zhang and Li, 2007)] or simple MOPs [as illustrated by MOEA/D-M2M and MOSOS/D (Liu et al., 2014; Ganesh et al., 2023)] with the help of weight vectors or reference points. Therefore, many improvements in this type of MOEAs focus on obtaining more appropriate weight vectors (reference points). For instance, Ma et al. (2020) propose an adaptive weight vector adjustment strategy, in which the weight vectors are periodically modified to enhance the searching capability of the algorithm. In MOEA/D-CSM (Liu et al., 2021), a dynamic reference points generation strategy, which considers the local knowledge in objective space, is proposed to obtain the reference points that can adapt well to MOPs with irregular Pareto fronts. In DMO-QPSO (You et al., 2021), a combination of the quantum-behaved particle swarm optimization (QPSO) algorithm and the MOEA based on decomposition (MOEA/D) is proposed. This integration aims to enable QPSO to effectively address MOPs while leveraging the strengths of QPSO. Additionally, the algorithm introduces some non-dominated solutions to guide other particles in the global best guidance group. The results indicate that the DMO-QPSO algorithm excels in addressing both two-objective and three-objective problems.

For index-based MOEAs, the additional indexes are adopted to determine the priority of solutions or guide the selection process in algorithms. Some representative indexes are hypervolume (HV) (While et al., 2006; Deist et al., 2023), inverted generation distance (IGD) (Zhou et al., 2006; Ishibuchi et al., 2019), dominance move(DoM) (Lopes et al., 2022), and R2 (Ma et al., 2018), and so on. In recent years, the hybrid index, which combines multiple indexes to improve search efficiency, has been proposed. For example, a hybrid index that combines HV and R2 has been adopted (Shang and Ishibuchi, 2020; Shang et al., 2020). Using HV to assess the distribution of the obtained Pareto fronts and R2 to measure the distance between these Pareto fronts and the ideal ones, the hybrid index facilitates algorithms in attaining a balance between convergence and population diversity.

### 2.3 Sparse MOEAs

Studies have revealed that numerous MOPs possess sparse Pareto optimal solutions, particularly those with large-scale decision variables (Tian et al., 2021b). Such MOPs featuring sparsity are commonly referred to as sparse multi-objective optimization problems (SMOPs). In other words, most decision variables of the Pareto optimal solutions in SMOPs are 0. In this case, traditional MOEAs can not obtain satisfactory results when solving SMOPs. This is because traditional MOEAs do not study the sparse distribution of Pareto optimal solutions and thus cannot effectively generate candidate solutions with sparsity in the evolution process.

In recent years, some variations of MOEAs have been applied to solving SMOPs successfully. These algorithms, called sparse multi-objective evolutionary algorithms (SMOEAs), can be divided into two categories. In the first type, SMOEAs adopte the dimension reduction techniques that are commonly used in machine learning. For example, to reduce the number of sparse large-scale decision variables, MOEA/PSL (Tian et al., 2021a) leverages a denoising auto-encoder (DAE) followed by the utilization of a restricted Boltzmann machine (RBM) for acquiring insight into the sparse distribution of decision variables. PM-MOEA (Tian et al., 2022) adopts pattern mining techniques to identify the maximal and minimal candidate sets of non-zero decision variables from the population and apply specialized genetic operators to these patterns to achieve dimensional reduction. SMEA (Tian et al., 2023) proposes an effective approach for addressing sparse large-scale multi-objective evolutionary problems. The algorithm optimizes the binary vectors of each solution to estimate the sparse distribution of optimal solutions and introduces a rapid clustering method for significantly reducing the dimensionality of the search space. This algorithm partitions a substantial number of decision variables into multiple groups, where all variables within the same group are collectively represented by a single variable for optimization. This innovative strategy substantially diminishes the search space, thereby enhancing the convergence speed.

The search efficiency has been improving for the first type of SMOEAs since the dimension of search space has been reduced. However, some dimension reduction techniques may need high computational cost, and there is no sparsity-related knowledge as guidance information in the evolution process of the algorithms. In the second type, SMOEAs combines the conventional framework of MOEAs (such as NSGA-II) and a hybrid encoding method of solutions. For example, S-NSGA-II (Kropp et al., 2023) introduces a novel set of evolutionary operators, which include Varied Striped Sparse Population Sampling (VSSPS), Sparse Simulated Binary Crossover (S-SBX), and Sparse Polynomial Mutation (S-PM), to address SLMOPs. The aforementioned operators demonstrate remarkable efficacy in solving SLMOPs, particularly when evaluated using HV. In SparseEA, as introduced by Tian et al. (2020), a solution is represented by two components: a real vector for the original decision variables, and a binary vector, often referred to as a “mask vector,” which governs the solution's sparsity. SparseEA2 adds a decision variable grouping strategy to accelerate the convergence speed of generating sparse Pareto optimal solutions. However, the decision variable grouping strategy in SparseEA2 is designed based on the random grouping method without considering the relation between variables. S-ECSO (Wang et al., 2022), an enhanced competitive swarm optimization approach, which adopts the strongly convex sparse operator(SCSparse), is designed to address SMOPs and exhibits outstanding performance.

As described in Section 1, the channel selection problems in BCIs is a typical SMOP. Based on the domain knowledge in the specific problem, this article proposes a two-stage sparse multi-objective optimization evolutionary algorithm, namely TS-MOEA. In TS-MOEA, both the sparsity and domain knowledge are considered in the design of the fundamental operators. The detailed description of TS-MOEA is shown below.

## 3 Method

### 3.1 Formulation of two-objective channel selection optimization problem in two stages

**Figure 3:**
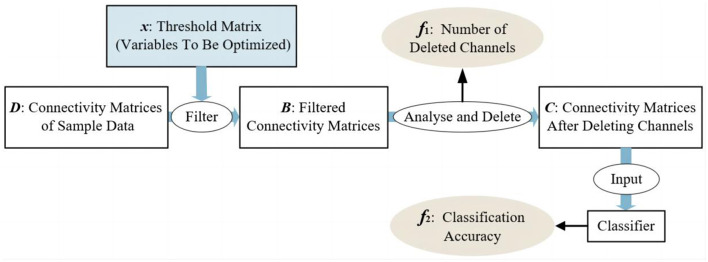
Channel selection problem.

This paper aims to select as few channels as possible with acceptable task accuracy. So, the number of deleted channels (*f*_1_) and the accuracy of tasks (*f*_2_) are the two maximized objectives that come to mind intuitively. Figure 3 illustrates the modeling process for the channel selection problem. Firstly, the raw signals are processed into sample data by computing the PCC values between each channel. Therefore, the sample data are all presented in the form of correlation matrices (as described in Section 2.1). Then, for the channel optimization problem, the threshold matrix *x* is considered as the decision variable that needs to be optimized. By filtering the sample data through the threshold matrix, it is easy to determine which channel can be deleted (Algorithm 1), and thus the value of *f*_1_ can be obtained, which is the number of deleted channels. Based on the channels that have been deleted, the subset of retained channels can be obtained. By using the data matrix of these selected/retained channels as the input for the classifier, the classification accuracy for a specific task can be achieved, denoted as *f*_2_. In summary, the channel optimization problem is modeled as a maximization two-objective problem. As shown in Figure 3, the threshold matrix *x* contains the decision variables that need to be optimized, and *x* has the same size as the connectivity matrices of sample data. Where *D* = {*D*_1_, *D*_2_, ..., *D*_*N*_} and *N* is the number of samples, *D*_*i*_(1 ≤ *i* ≤ *N*) is the correlation matrix for the *i*^*th*^ sample. After filtering *D* by *x*, one can obtain the set of the filtered correlation matrices, denoted as *B*, for all samples. Specifically, *B* = {*B*_1_, *B*_2_, ..., *B*_*N*_} and *B*_*i*_(1 ≤ *i* ≤ *N*) is the filtered correlation matrix for the *i*^*th*^ sample. Then, the channels to be deleted can be determined by analyzing the filtered correlation matrices, thereby obtaining the number of selected channels (*f*_1_). The detailed procedure of obtaining the number of deleted channels, i.e., *f*_1_, is given in Algorithm 1. As shown in Algorithm 1, if a channel is irrelevant to most channels, then this channel is most likely useless for the specific task and will be deleted (Lines 9, 10). In Line 10 of Algorithm 1, The value of *s* determines the difficulty level for channels to meet the deletion criteria. The correlation matrix after channel deletion *C* = {*C*_1_, *C*_2_, ..., *C*_*N*_} can be obtained based on *B*. For the *k*^*th*^ sample, if the *j*^*th*^ channel can be deleted, then *C*_*k*_ can be acquired by setting the elements in both the *i*^*th*^ row and the *k*^*th*^ column of *B*_*k*_ to 0 (Lines 17, 18). After that, *C* will be used as the input of classifiers, and then the accuracy of classification tasks (*f*_2_) can be obtained. The above-mentioned two-objective optimization problem can be formulated as shown in Equation (5). Please note that any classifier can be utilized for obtaining classification results. Since the focus of this paper does not center on the classifier itself, the classic support vector machine (SVM) is selected here. The hyperparameters used in SVM are obtained through the grid search method, in conjunction with 5-fold cross-validation. The hyperparameter determination process begins with establishing a range of potential values for each hyperparameter, forming a parameter grid. After evaluating each set of hyperparameter combinations by the 5-fold cross-validation method, the best values for the hyperparameters adopted in SVM will be obtained. The classification accuracy of SVM using the best hyperparameter swill regarded as *f*_2_.


(5) $$\begin{array}{l} Maximum\;F(X)=(f_{1}(X),f_{2}(X))\\ f_{1}(X)=m_{d},m_{d}\;is\;the\;number\;of\;deleted\;channels\\ f_{2}(X)=classifer(C) \end{array}$$

(6) $$\begin{array}{l} \begin{array}{c} f_{1}^{*}\left(X\right)=0.5*\frac{zero\left(C\right)}{N_{C}}+0.5*\frac{m_{d}}{m} \end{array} \end{array}$$

(7) $$\begin{array}{l} Maximum\;F(X)=(f_{1}^{*}(X),f_{2}(X))\\ f_{1}^{*}(X)=0.5*\frac{zero(C)}{N_{C}}+0.5*\frac{m_{d}}{m}\\ f_{2}(X)=classifer(C) \end{array}$$

**Algorithm 1:**
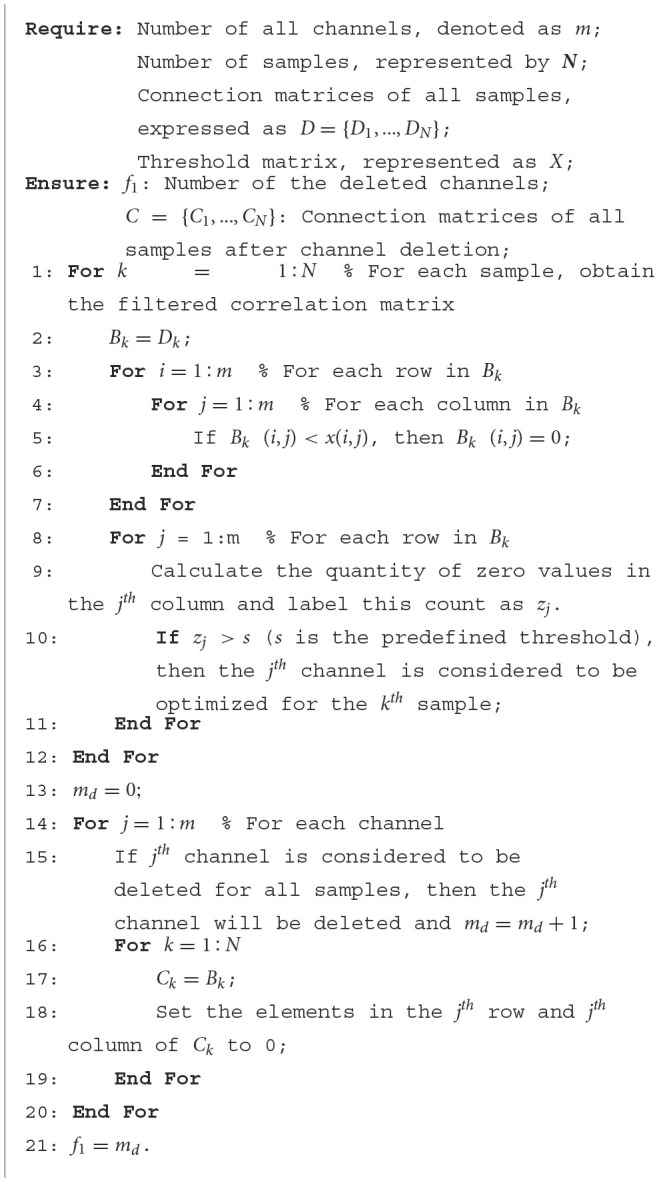
The detailed procedure of obtaining the number of deleted channels.

**Figure 4:**
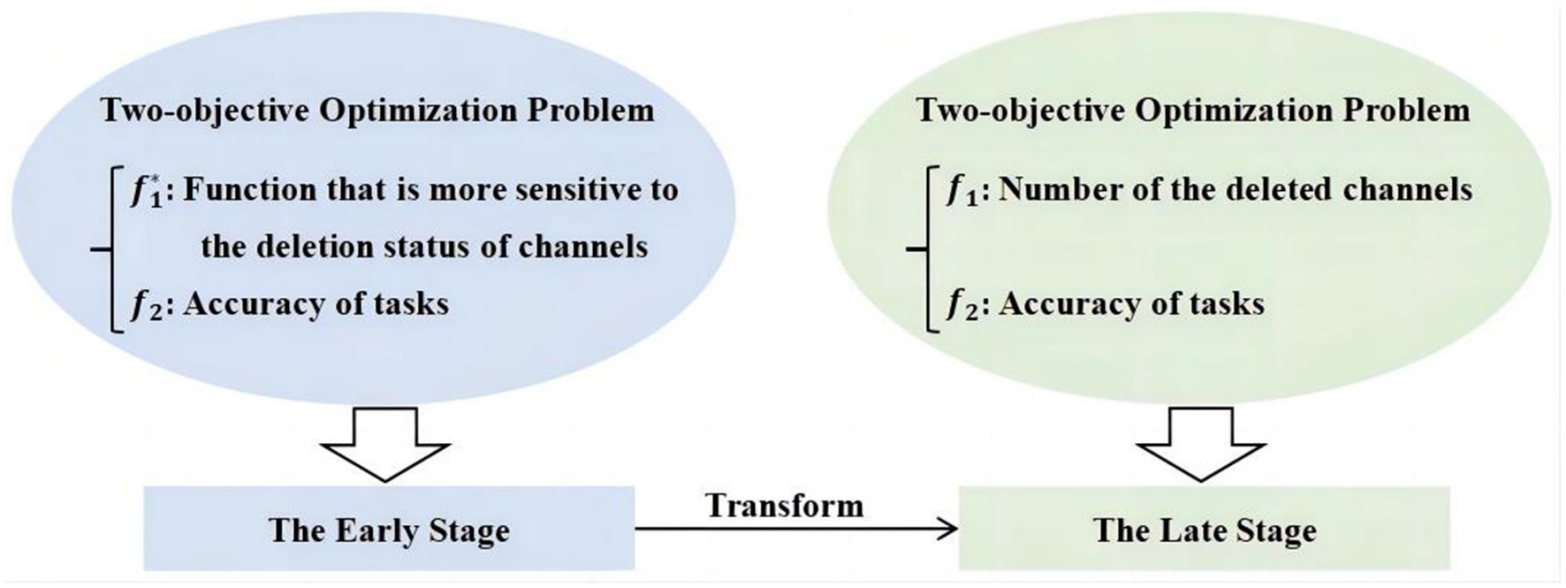
Illustration of two-stage framework.

However, as shown in Algorithm 1, a channel can only be deleted if it is unanimously agreed upon by all samples. Particularly, when *s* is set to a large value, meeting the deletion criteria for channels becomes even more challenging. This difficulty results in MOEAs encountering stagnation when optimizing *f*_1_ (number of deleted channels). To address this problem, a novel objective function $f_{1}^{*}$ is introduced, which offers higher sensitivity in reflecting the deletion status of channels. As expressed in Equation (6), $\frac{zero\left(C\right)}{N_{C}}$ signifies the ratio of zero elements in *C*. Specifically, *zero*(*C*) represents the count of zero elements, and *N*_*C*_ is the total number of elements in *C*. Let *m*_*d*_ denote the number of deleted channels and *m* denote the total count of channels. Then, $\frac{m_{d}}{m}$ represents the proportion of deleted channels to the total channels. In this case, the multi-objective problem can be formulated as shown in Equation 7. In Equation 7, the number of deleted channels (*f*_1_) from the original Equation (5) is transformed into $f_{1}^{*}$, which represents the weighted average sum of the proportion of zero elements in *C* and the proportion of deleted channels to the total. After this transformation, the first objective function in the two-objective optimization model has shifted from a discrete integer search space to a continuous real number search space, which reduces the risk of the algorithm falling into a locally optimal solution. Therefore, compared to the two-objective problem model in Equation (5), the model in Equation (7) is more sensitive to the deletion status of channels, rendering it less susceptible to stagnation. Hence, this paper introduces a two-stage framework, as illustrated in Figure 4, employing different two-objective problem models in the early and late stages of the proposed algorithm.

### 3.2 Framework of TS-MOEA

**Figure 5:**
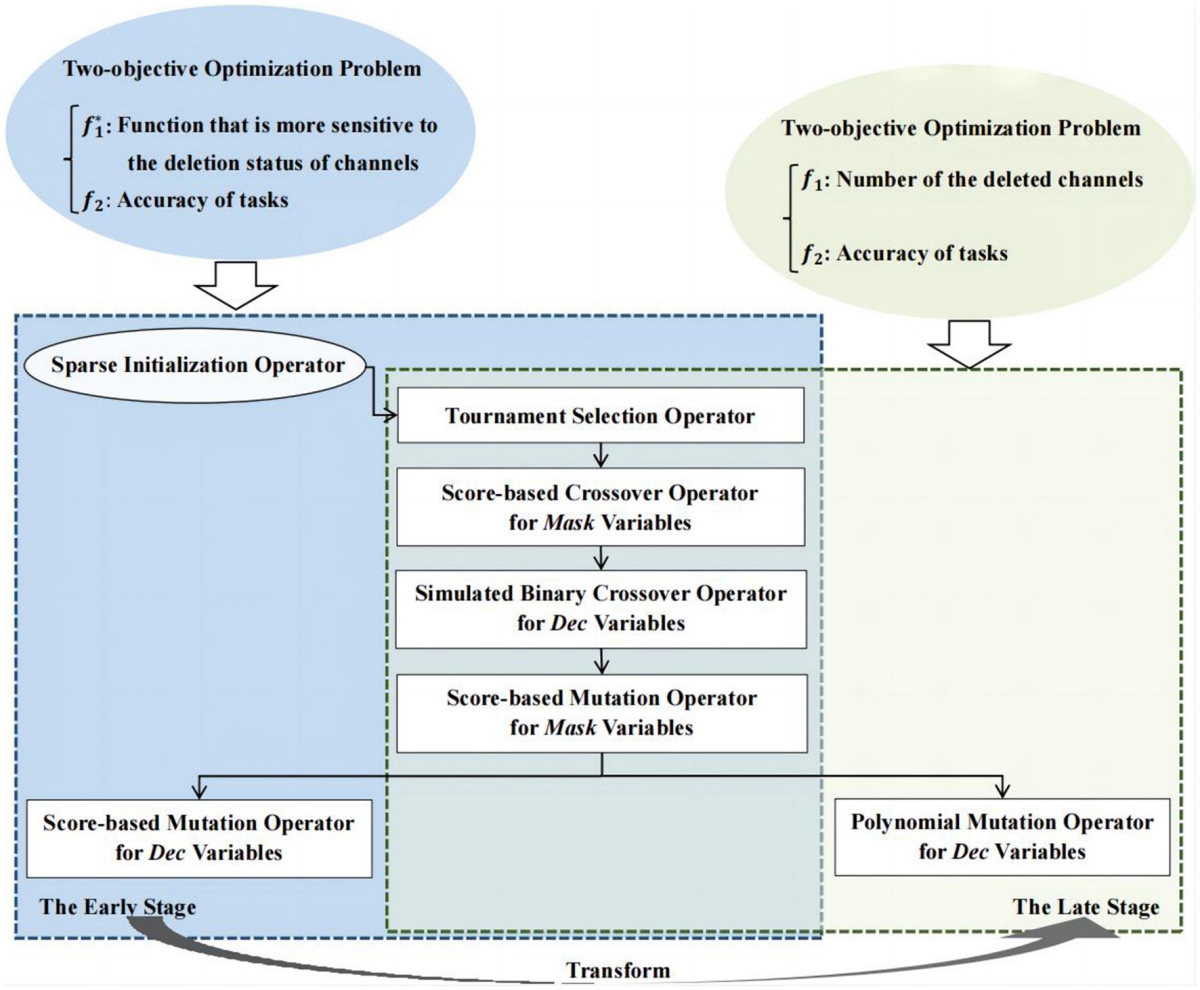
Framework of TS-MOEA.

In this paper, a two-stage sparse multi-objective evolutionary algorithm, named TS-MOEA, is introduced to address channel selection problems in BCIs. As illustrated in Figure 5, TS-MOEA adopts a two-stage framework comprising the early and late stages, each dedicated to distinct optimization problem models. It also can be observed from Figure 5 that the early and late stages share most operators. Specifically, in addition to the sparse initialization operator, the only difference between the two stages is the mutation of *Dec* variables. Furthermore, due to the sparsity of the correlation matrix, TS-MOEA adopted a hybrid representation of decision variables, which contains *Dec* variables (real numbers) and *Mask* variables (binary numbers). Algorithm 2 gives the detailed procedure of TS-MOEA.

**Algorithm 2:**
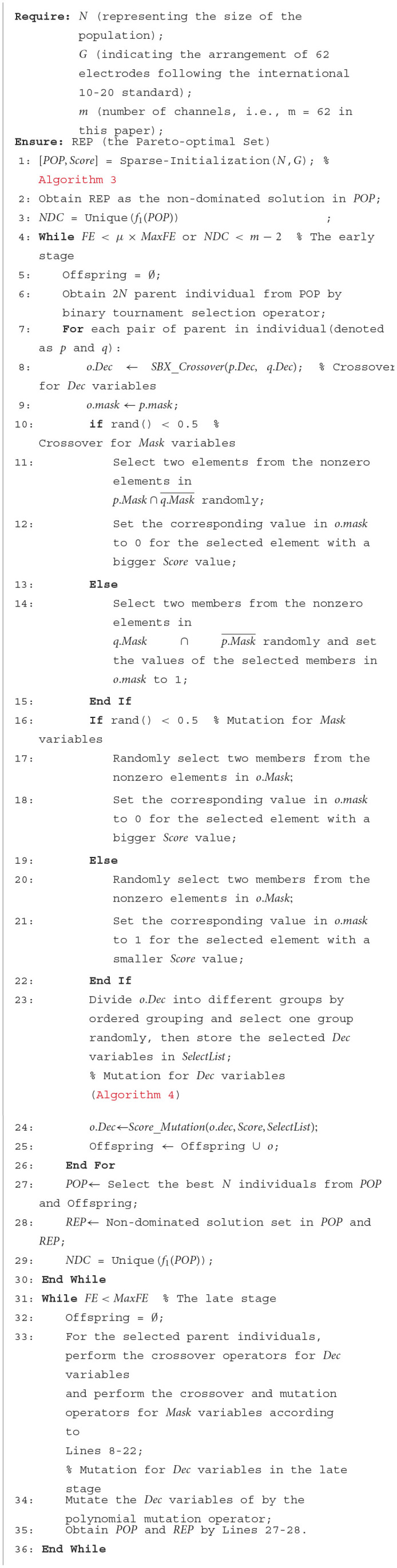
Procedure of TS-MOEA.

In TS-MOEA, the output population obtained in the first stage becomes the input population for the late stage. To ensure population diversity in the late stage, TS-MOEA adopts a transformation condition between the two stages, which takes into consideration both the total of consumed function evaluations (*FE*) and (*NDC*), as shown in Line 4 of Algorithm 2. *NDC* represents the number of different *f*_1_ values obtained by *POP*. To ensure population diversity in the late stage, TS-MOEA adopts a transformation condition between the two stages, which takes into consideration both *FE* and *NDC*, as shown in Line 4 of Algorithm 2. Where *FE* is the current number of function evaluations consumed by the algorithm and *NDC* represents the number of different *f*_1_ values obtained by *POP*. Since *f*_1_ is one of the objective functions optimized by TS-MOEA in the late stage, a larger *NDC* implies that the population exhibits better diversity in the late stage of TS-MOEA. If the number of deleted channels is equal to *m*, i.e., all channels are removed, this is nonsensical. Moreover, since TS-MOEA is designed based on the correlation matrix between channels, this implicitly presupposes that the number of retained channels is greater than or equal to 2. Therefore, the possible values for the number of deleted channels can be any integer within the range [0, *m*−2]. When the *NDC* is *m*−2, it indicates that the population generated in the first stage is sufficiently diverse to serve as the input population for the next stage. Moreover, if the number of the consumed function evaluations of the early stage exceeds the preset threshold, i.e., μ × *MaxFE*, the algorithm can also transfer from the early stage to the late stage. In this case, μ controls the transformation between the two stages, and its value has been investigated in detail in Section 4.2.

TS-MOEA introduces a sparse initialization operator to generate the initial population for channel selection problems (Line 1 in Algorithm 2). In the sparse initialization operator, each decision variable will be assigned a *Score* value, which is calculated according to the problem-domain knowledge. The detailed description of the sparse initialization operator is given in Section 3.3. Both the early and late stages in TS-MOEA adopt the binary tournament selection operator (Lavinas et al., 2018) to obtain parent individuals (Line 6). The crossover and mutation for *Dec* and *Mask* variables utilize different strategies. Specifically, the simulated binary crossover operator (Deb and Beyer, 2001; Zhassuzak et al., 2024) is adopted for *Dec* variables (Line 8), while the *Score*-based crossover operator, which is inspired by SparseEA2, is utilized for *Mask* variables (Lines 10–15). The mutation for *Mask* variables is implemented by the *Score*-based mutation operator as shown in Lines 16–22. To balance convergence and population diversity, a *Score*-based mutation and the conventional polynomial mutation operators are utilized For *Dec* variables in the early and late stages of TS-MOEA, respectively (Lines 24, 34). The description of the proposed *Score*-based mutation operator has been given in Section 3.4. TS-MOEA utilizes the sequential grouping strategy (Zille et al., 2016) to divide decision variables into groups. Specifically, if it is required to split *d* decision variables into *k* groups, then the first [*d*/*k*] decision variables will be classified into the first group, the next [*d*/*k*] decision variables will be classified into the second group, and so on. Here, [*d*/*k*] denotes the integer closest to *d*/*k*. Since the binary tournament selection, simulated binary crossover, and polynomial mutation operators are widely adopted in various MOEAs, their details will not be presented here to save space.

### 3.3 Sparse initialization operator

**Figure 6:**
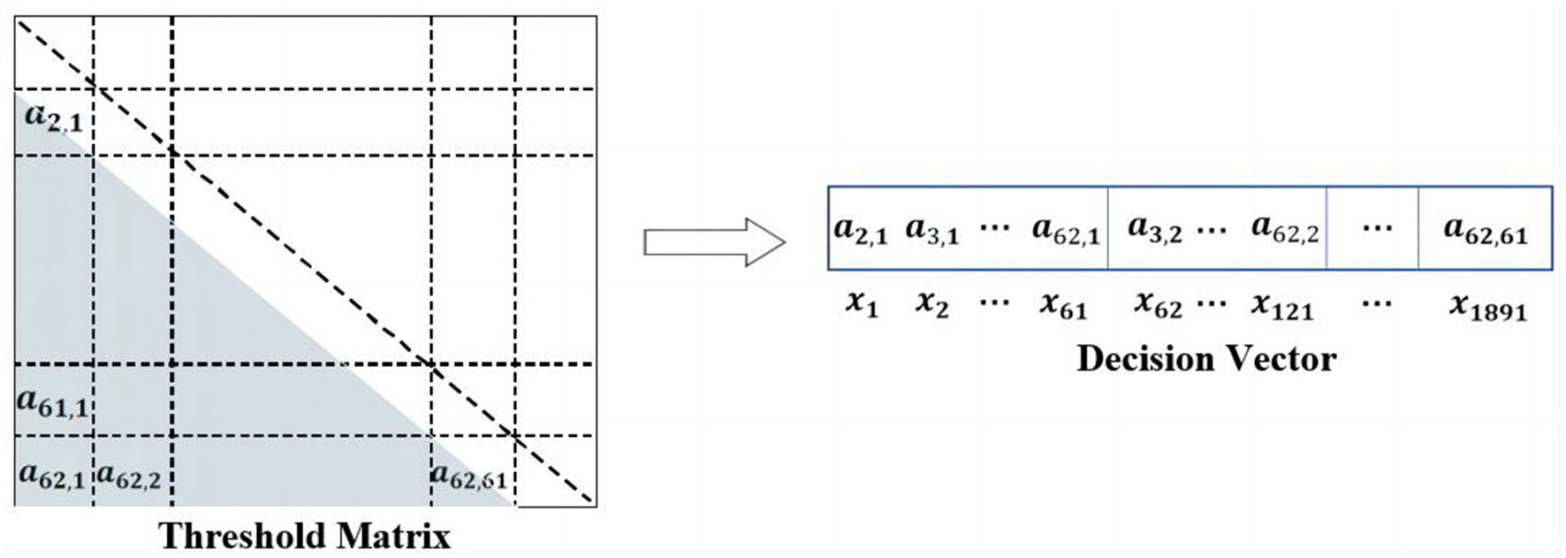
Illustration of decision vector.

**Figure 7:**
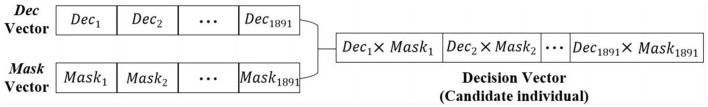
Hybrid representation of decision vector.

In the proposed TS-MOEA, the threshold matrix *x* is the optimization target, as shown in Figure 3. *x* is employed to filter the correlation matrix of samples. Since the correlation matrix is symmetric, *x* will be rearranged as a decision vector. For instance, in this paper, the correlation matrix is 62 × 62 due to the utilization of 62 channels. Therefore, the size of the decision vector will be 1 × 1, 891, as shown in Figure 6. Inspired by SparseEA2, this paper adopted a hybrid representation of decision variables, which contains real variables (*Dec* vector) and binary variables (*Mask* vector). As illustrated in Figure 7, both the *Dec* vector and the *Mask* vector share the same size as the decision vector. The actual decision vector is obtained by multiplying corresponding elements from the *Dec* and *Mask* vectors.

Algorithm 3 provides a detailed procedure of the proposed sparse initialization operator. In this operator, the first step is to calculate the *Score* value of each variable in the decision vector (Lines 2–6). The *Score* values will later be used to determine whether elements in the *Mask* vector should be set to 0. Research has revealed that the relationship between brain regions relates to their location and length from each other (van den Broek et al., 1998; Reznik and Allen, 2018). Therefore, the calculation of *Score* values in this paper is based on domain-specific knowledge, which includes the location of channels and the distances between channels.

**Algorithm 3:**
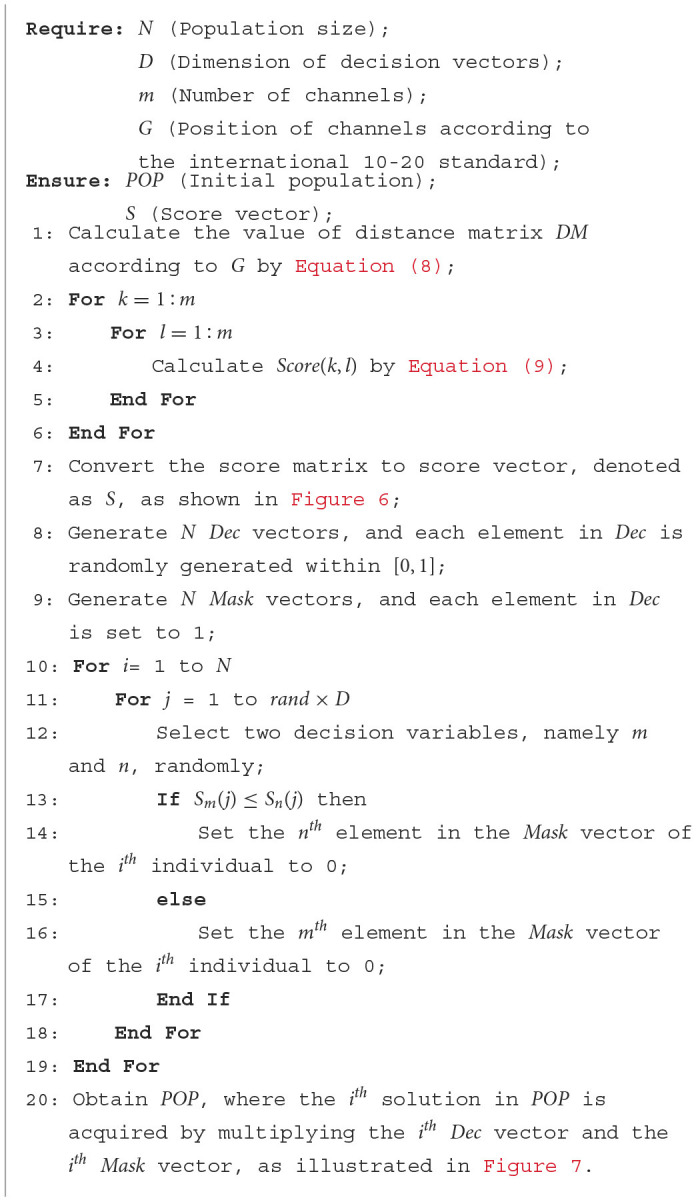
Sparse initialization operator.

The calculation of *Score* values of the decision variables are given in Equations (8), (9). As presented in Equation (8), *G*_*Channe*_*l*__*k*__ represents the position of channel *k*, which can be acquired according to the international 10–20 standard. ||*G*_*Channe*_*l*__*k*__−*G*_*Channe*_*l*__*l*__||_2_ signifies the Euclidean distance between *G*_*Channe*_*l*__*k*__ and *G*_*Channe*_*l*__*l*__. In Equation (9), *Max*(*DM*) and *Min*(*DM*) denote the maximum and minimum distances between channels. *Location*_*k*_ = 1 denotes that *Channel*_*k*_ is located in the left hemisphere, while *Location*_*k*_ = –1 indicates that *Channel*_*k*_ is located in the right hemisphere, respectively. If *Channel*_*k*_ is positioned in the inter-hemispheric junction area of the brain, as illustrated by the dotted circles in Figure 2, then *Location*_*k*_ will be set to 0. As indicated in Equation (9), two channels located in different cerebral hemispheres have higher *Score* values compared to channels situated in the same hemisphere. Additionally, channels that are farther apart have higher *Score* values. In Equation (9), *R* is the preset channel radius, whose value has been investigated in Section 4.3. For *Channel*_*k*_ and *Channel*_*l*_, the larger the *Score* value, the easier it is for the corresponding element in *Mask* to be 0 (Lines 13–17) and the easier it is for the correlation coefficient of *Channel*_*k*_ and *Channel*_*l*_ to be 0 after filtering.


(8) $$DM(k,l)={\left\|G_{Channel_{k}}-G_{Channel_{l}}\right\|}_{2}$$

(9) $$\begin{array}{l} Score(k,l)\\ =\{\begin{array}{ll} \frac{DM(k,l)-R}{2(Max(DM)+R)} & if\;Location_{k}=Location_{l}\\ \frac{DM(k,l)}{2(Max(DM)+R)} & if\;Location_{k}\neq Location_{l}\;and\;DM(k,l)<R\\ \frac{DM(k,l)+R}{2(Max(DM)+R)} & if\;Location_{k}\neq Location_{l}\;and\;DM(k,l)\geq R \end{array} \end{array}$$

### 3.4 *Score*-based mutation operator

As explained in Section 3.2, the output population from the early stage in TS-MOEA serves as the input population for the late stage. Hence, the quality of the population acquired in the early stage significantly impacts the ultimate performance of the proposed TS-MOEA. To effectively leverage the problem-domain knowledge to steer the search process of TS-MOEA, a *Score*-based mutation operator, which utilizes the *Score* values of decision variables, is introduced for *Dec* vectors in the early stage of TS-MOEA. Algorithm 4 presents the detailed procedure of the *Score*-based mutation operator.

**Algorithm 4:**
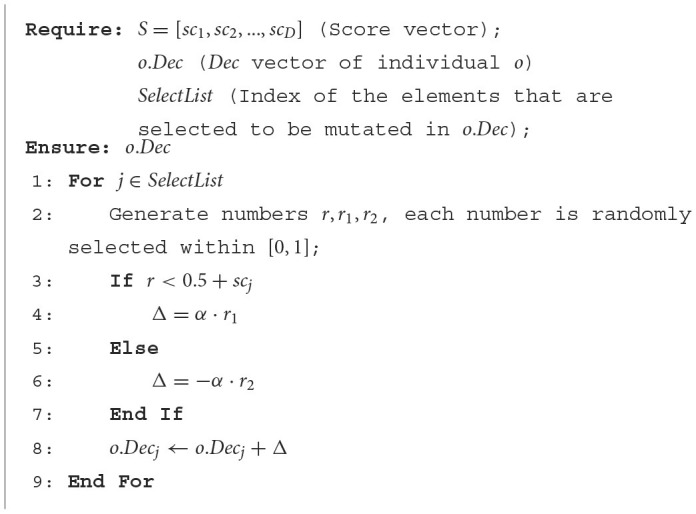
Score-based mutation operator.

For *o*.*Dec* (the *Dec* vector of individual *o*), if *sc*_*j*_ is large, i.e., the corresponding two channels are close to each other, then *o*.*Dec*_*j*_ will have a greater probability to be a large value after mutation (Lines 4, 8 in Algorithm 4). In this case, the two channels that are related to *Dec*_*j*_ tend to be regarded as uncorrelated after filtering (Line 5 in Algorithm 4). In Algorithm 4, α controls the magnitude of mutation and is set to 0.1 empirically.

## 4 Results

### 4.1 EEG data and parameter settings

The unprocessed EEG signals were gathered from a group of 9 participants aged between 21 and 30 during a fatigue detection task. Throughout the data collection phase, participants underwent a wake-sleep-wake cycle post-lunch, a period when many people typically experience fatigue symptoms. For experiment integrity, all volunteers were required to wake up before 8:30 a.m. and abstain from alcohol and drugs. During the fatigue detection task, participants lay on a bed with closed eyes, responding to auditory cues via their headsets by promptly opening their eyes. Volunteers were considered alert if they responded within 2 seconds; otherwise, they were classified as fatigued. Further details regarding the processing of the acquired EEG signals are elaborated in Section 2.1.

This paper utilizes the Hypervolume (HV) metric, as described by While et al. (2006). To evaluate the efficiency of the proposed algorithm. HV measures an algorithm's convergence and diversity by calculating the hypercube's volume, which is formed by the non-dominated solutions obtained by the evaluated algorithm and a predetermined reference point. The reference point is typically chosen to be worse than the function values of each solution in the current evaluated solution set. Therefore, a greater HV value signifies better performance of the assessed algorithm. Since the optimization problem in this paper is a two-objective problem that requires maximization, the reference point *z* is generated according to the following Equation (10). Where *P* represents the Pareto-optimal-set obtained by the evaluated algorithm.


(10) $$\begin{array}{l} z=(z_{1},z_{2})\\ z_{1}=\underset{x\in P}{\min}f_{1}(x)-0.1\\ z_{2}=\underset{x\in P}{\min}f_{2}(x)-0.1 \end{array}$$

As depicted in Figure 6, when conducting channel selection for a BCI system comprising 62 channels, the number of the decision variables is 1,891. Consequently, the channel selection problem addressed in this paper qualifies as a large-scale MOP. Moreover, owing to the sparsity of the correlation matrix of channels, the channel selection problem discussed in this paper also falls within the category of sparse large-scale MOPs. To assess the effectiveness of the proposed algorithm, TS-MOEA is compared with several advanced large-scale MOEAs, containing SpaseEA2 (Zhang et al., 2021), SLMEA (Tian et al., 2023), S-ECSO (Wang et al., 2022), CMMO (Ming et al., 2023), and S-NSGA-II (Kropp et al., 2023). Among these comparison algorithms, SparseEA2 is an effective sparse multi-objective optimization algorithm, whereas S-NSGA-II and S-ECSO are specialized for large-scale multi-objective optimization tasks. CMMO, a newly introduced algorithm, excels in finding an optimal equilibrium between diversity and convergence for multi-objective optimization problems. SLMEA is specialized for super-large-scale sparse multi-objective problems. For fair comparisons, all algorithms adopt the maximum number of function evaluations (*MaxFE*) of 20000 and the population size (*N*) of 200. The detailed settings of algorithms are given in Table 1, and *D* is the number of decision variables.

**Table 1:** Algorithm configuration parameters.

**Algorithm**	**Parameter setting**	**References**
SparseEA2	The crossover probability is 1, while the mutation probability is 1/*D*;	Zhang et al., 2021
	Both crossover and mutation have a distribution index of 20.	
	SLMEA	The crossover probability is 1, while the mutation probability is 1/*D*;	Tian et al., 2023
	Both crossover and mutation have a distribution index of 20.	
	S-ECSO	Inertia weight *w* is 0.7968; μ_initial_ is set to 0.35;	Wang et al., 2022
	The learning factors *C*_1_ and *C*_2_ are both assigned the value of 1.4962.	
	CMMO	The crossover probability is 1, while the mutation probability is 1/*D*;	Ming et al., 2023
	Both crossover and mutation have a distribution index of 20; μ, τ and θ are set to 0.1.	
	S-NSGA-II	The crossover probability is 1, while the mutation probability is 1/*D*;	Kropp et al., 2023
	Both crossover and mutation have a distribution index of 20.	
		The crossover probability is 1, while the mutation probability is 1/*D*;	
TS-MOEA	Both crossover and mutation have a distribution index of 20.	
	*R*, μ and *s* are set to 0.1, 0.2 and 40, respectively.	

### 4.2 Statistical results and analysis

Table 2 provides the statistical results of all six algorithms over 30 independent runs, measured in terms of HV. In this table, the best average HV values are highlighted in bold. Symbols “+,” “–,” and “≈” indicate that, according to the Wilcoxon rank-sum test (Yaman et al., 2021) at a 5% significance level, the performance of the compared algorithm is significantly better than, worse than, or similar to that of the proposed TS-MOEA, respectively.

**Table 2:** Average HV values achieved by TS-MOEA and other comparative MOEAs.

**Subject**	**SparseEA2**	**SLMEA**	**S-ECSO**	**CMMO**	**S-NSGA-II**	**TS-MOEA**
1	2.79E+01 -	4.46E+00 -	1.36E+01 -	8.86E+00 -	5.13E+00 -	**6.16E+02**
2	2.42E+01 -	1.25E+01 -	1.81E+01 -	2.15E+01 -	1.16E+02 -	**6.63E+02**
3	4.51E+01 -	7.01E+00 -	1.99E+01-	1.26E+01 -	1.17E+02 -	**7.41E+02**
4	2.11E+01 -	4.63E - 01 -	9.35E+00 -	6.58E+01 -	1.04E+02 -	**4.29E+02**
5	2.93E+01 -	3.02E+01 -	2.42E+01 -	2.03E+01 -	3.13E+01 -	**7.33E+02**
6	2.21E+01 -	1.30E+01 -	9.70E+00 -	7.36E+00 -	1.32E+01 -	**6.28E+02**
7	2.02E+01 -	9.73E+00 -	1.13E+01 -	6.21E+00 -	1.68E+01 -	**7.27E+02**
8	2.73E - 01 -	9.99E+00 -	5.20E+01 -	1.44E+00 -	1.13E+02 -	**3.81E+02**
9	2.08E+01 -	1.28E+01 -	1.13E+01 -	5.13E+00 -	1.06E+02 -	**5.37E+02**
+/-/≈	0/9/0	0/9/0	0/9/0	0/9/0	0/9/0	

Table 2 presents the statistical HV values obtained by TS-MOEA and other MOEAs. The numbers in bold are the best results achieved by algorithms and bold numbers in other tables also indicate the best results. The primary distinction between TS-MOEA and the comparative algorithms lies in TS-MOEA's utilization of a two-stage framework. Within this framework, the early stage is focused on discovering a diverse and well-distributed population. This is achieved by employing a two-objective problem model that is highly sensitive to the deletion status of channels. The late stage in TS-MOEA directly uses the number of deleted channels as an optimization objective, thereby striking a balance between the number of deleted channels and task accuracy. Furthermore, domain-specific knowledge is utilized to guide the evolutionary process in TS-MOEA. It can be observed from Table 2 that TS-MOEA outperforms other algorithms in terms of HV for all 9 subjects, which indicates the effectiveness of the proposed two-stage framework.

**Figure 8:**
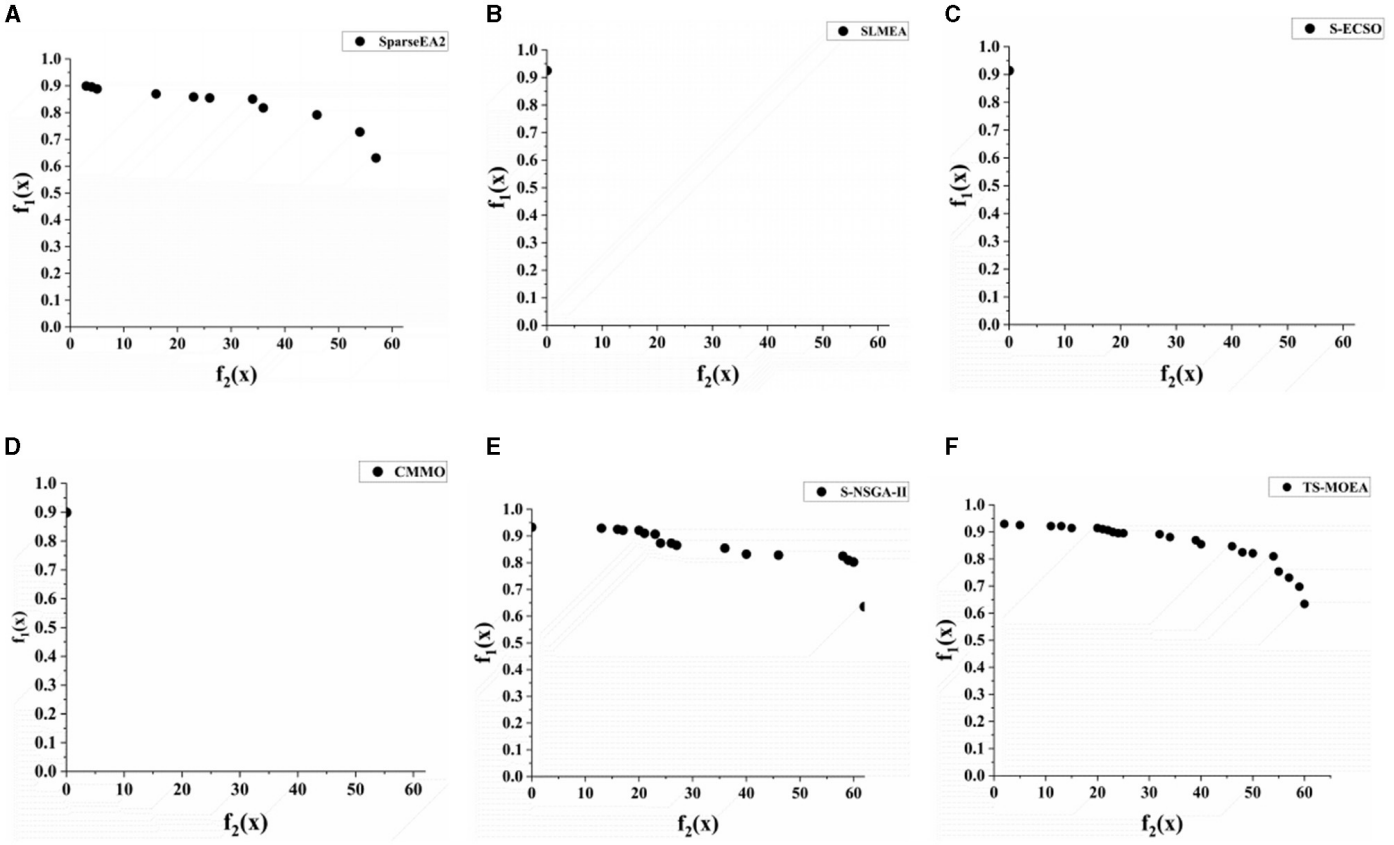
Pareto frontiers generated by all algorithms for Subject 2. **(A–F)** give the Pareto fronts generated by SparseEA2, SLMEA, S-ECSO, CMMO, S-NSGA-II, and TS-MOEA, respectively.

Among the five comparative algorithms, there are algorithms that are specifically designed for sparse large-scale optimization problems. However, these algorithms still perform worse than the proposed algorithm for the channel selection problem. This is mainly because none of these comparative algorithms utilize knowledge related to the problem domain. The statistical results indicate that incorporating domain-specific knowledge into algorithms can effectively enhance their performance when solving specific problems. Figure 8 displays the Pareto fronts generated by all algorithms for Subject 2. In Figure 8, it is evident that compared to other algorithms, TS-MOEA obtains the best Pareto front, which also verifies the efficiency of the proposed algorithm.

Table 3 gives the average classification accuracies achieved by SVM using all channels and partial channels selected by TS-MOEA for all subjects. Since TS-MOEA provides a set of Pareto optimal solutions, which includes a variety of different electrode selection schemes. To save space, Table 3 displays several representative channel selection schemes along with their corresponding classification accuracies. As can be seen from Table 3, the classification accuracy decreases as the number of selected channels decreases. However, in some cases where only a subset of channels is chosen (such as selecting 60 or 52 channels), the classification accuracy is either better than or slightly lower than when all channels are selected. This indicates that the proposed TS-MOEA can effectively reduce the number of channels used in BCIs while maintaining acceptable classification accuracy.

**Table 3:** Average classification accuracies achieved by SVM using all channels and partial channels selected by TS-MOEA.

**SVM using channels selected by TS-MOEA**		**SVM using all channels**
**Number of selected channels**	**Accuracy rate**	
60	98.62%	98.53%
52	95.18%	
42	88.02%	
32	80.55%	
22	70.27%	
12	57.09%	
2	53.23%	

For further comparison of the proposed TS-MOEA with other state-of-the-art MOEAs, including SparseEA2, SLMEA, S-ECSO, CMMO, and S-NSGA-II, Table 4 presents the average classification accuracies achieved for all subjects based on the varying number of channels selected by the algorithm. It can be observed from Table 4, SLMEA, S-ECSO, CMMO fail to provide classification accuracies in most cases. This is due to the poor diversity of these three algorithms (which can also be observed in Figures 8B–D), which results in their inability to obtain the Pareto-optimal solutions for the corresponding number of selected channels. SparseEA2 and S-NSGA-II are capable of obtaining well-distributed Pareto optimal solution sets, similar to the proposed TS-MOEA. However, in terms of classification accuracy, TS-MOEA achieves the best results. Therefore, the statistical results in Table 4 validate that the proposed algorithm indeed strikes a good balance between classification accuracy and the number of selected channels compared to the algorithms under comparison.

**Table 4:** Average classification accuracies obtained by all algorithms.

**Number of selected channels**	**SparseEA2**	**SLMEA**	**S-ECSO**	**CMMO**	**S-NSGA-II**	**TS-MOEA**
60	91.50%	97.48%	-	-	60.36%	**98.62%**
52	76.78%	-	-	-	58.22%	**95.18%**
42	60.37%	-	-	-	56.47%	**88.02%**
32	56.92%	-	-	-	56.47%	**80.55%**
22	56.47%	-	-	-	52.19%	**70.27%**
12	55.40%	-	-	-	51.43%	**57.09%**
2	45.98%	-	-	-	51.43%	**53.23%**

**Figure 9:**
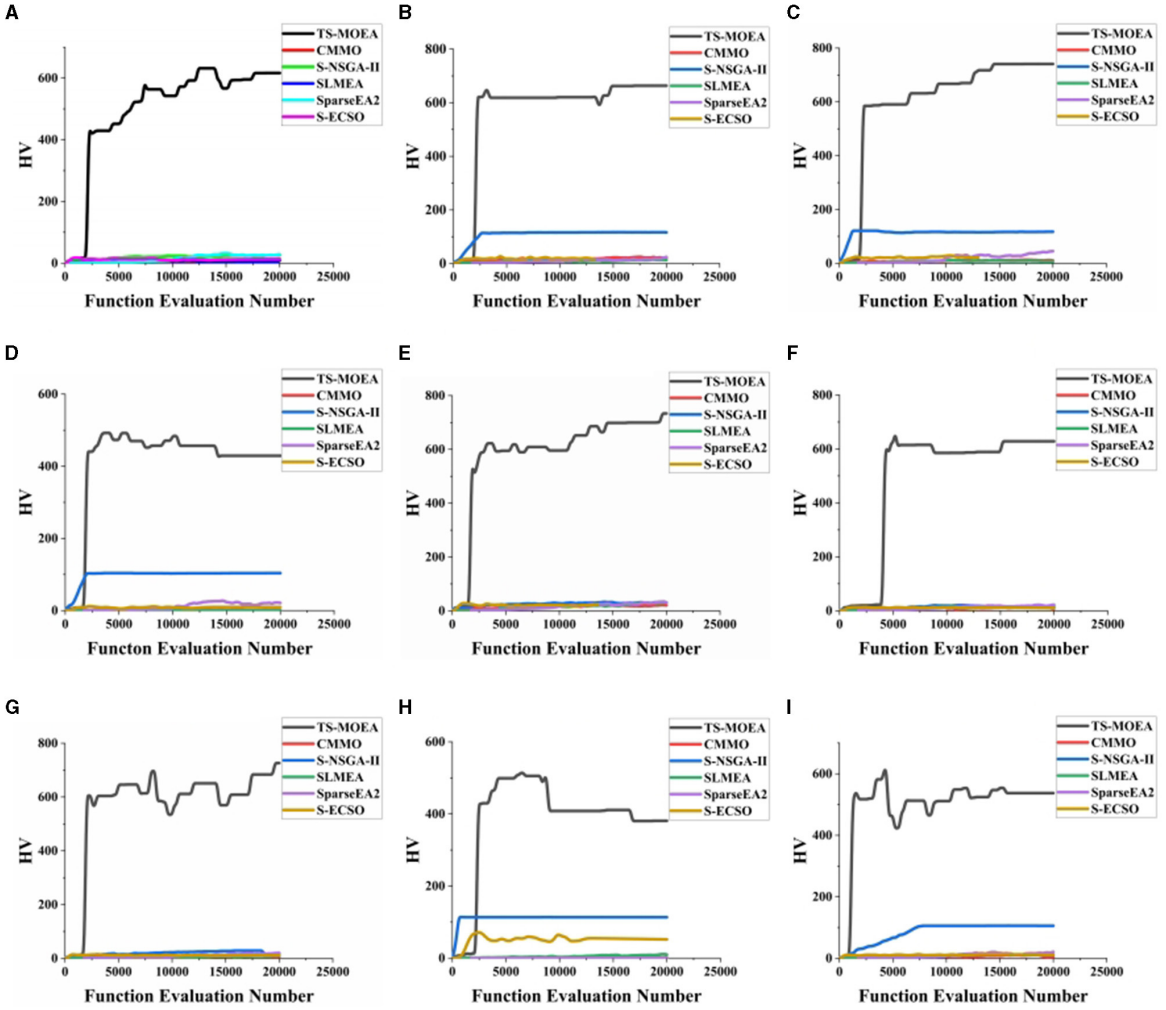
Convergence of 6 algorithm on 9 subjects. **(A–I)** demonstrate the convergence of all algorithms for Subject 1–Subject 9, respectively.

Figure 9 demonstrates the convergence of six comparative algorithms on 9 subjects. It can be observed from Figure 9 that TS-MOEA exhibits faster convergence compared to the other algorithms. This is mainly because the problem model adopted in the early stages of TS-MOEA can effectively prevents stagnation in the search process. Additionally, the sparse initialization and *Score*-based mutation operators can also accelerate the convergence speed of TS-MOEA.

In summary, there are two reasons for the superior performance of the proposed algorithm. First, TS-MOEA adopts a two-stage framework with different optimization model for each stage, which maintains population diversity and avoids premature maturity of the algorithm. It can be observed from Figure 8 that TS-MOEA has obtained a Pareto front with a better distribution. Second, in TS-MOEA, operators related to the problem domain are used to improve the effectiveness of the searching process. Specifically, when assigning scores to each decision variable, the position of the channels and the distance between them are considered. As shown in Figure 9, TS-MOEA demonstrates the best convergence, which also indicates the effectiveness of the domain-related operators used in TS-MOEA.

**Figure 10:**
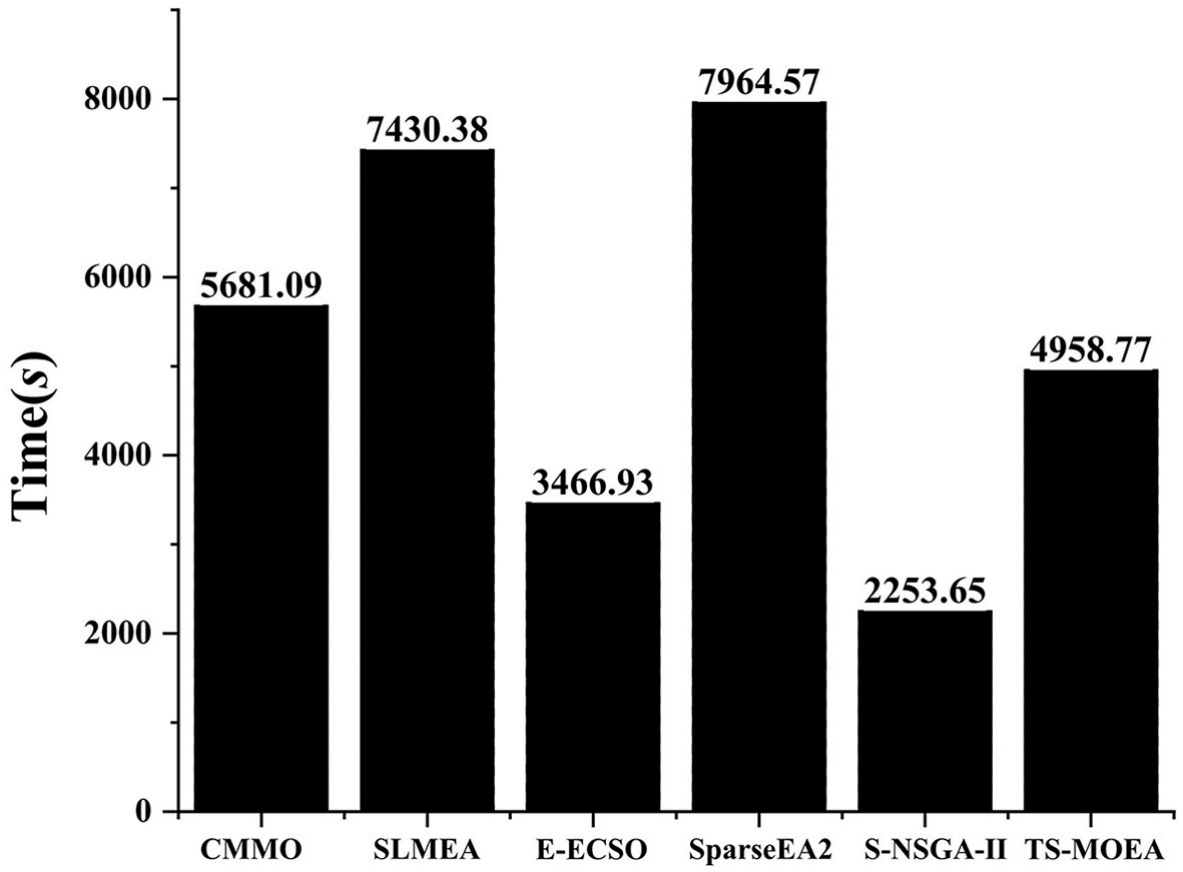
Average running times for all algorithms on Subject 1.

Figure 10 presents the average execution time of all algorithms tested on Subject 1. It can be found from Figure 10 that S-NSGA-II has the least running time, followed by E-ECSO and the proposed TS-MOEA. SparseEA2 has the highest average running time. This is because the strategy used in SparseEA2 to obtain the *Score* values of decision variables incurs a increased computational cost, especially when there are a significant amount of decision variables. In S-NSGA-II, operators designed for efficient handling of large-scale sparse multi-objective optimization problems are introduced. These operators enable S-NSGA-II to achieve high efficiency when dealing with channel selection problems with a significant amount of decision variables. For S-ECSO, the algorithm employs a three-party competition mechanism to guide its evolutionary process. Compared to commonly used genetic operators such as crossover and mutation, the three-party competition mechanism is simpler and consumes less computational cost.

### 4.3 Statistical results and analysis on the DEAP dataset

The DEAP (Koelstra et al., 2011) dataset was collected from a group of 32 participants, specifically for human emotion recognition. The dataset consisted of 32 channels of EEG signals and 8 channels of peripheral physiological signals (PPS.) The signals were sampled at a rate of 512 Hz. During the data collection process, the participants watched 40 1-min music videos while their physiological signals were recorded. The data set for each trial consisted of a 3-second pre-trial time and a 60-second video viewing trial time. At the end of the trial, participants self-assessed themselves based on arousal, sense of worthiness, dominance, and likability, using discrete 9-point scales for each dimension. In this section, the arousal dimension from the DEAP dataset has been utilized as a categorical label for binary classification to validate the effectiveness of the proposed algorithm.

Table 5 demonstrates the average classification accuracy of the proposed algorithm on DEAP. As can be seen from Table 5, when using 30, 22, 17, and 12 channels, the average classification accuracy is close to the classification accuracy using all channels, which further demonstrates the effectiveness of the proposed algorithm. At the same time, the results also show that channel selection is not simply a matter of reducing the number of channels to improve the classification performance, and that it is necessary to find the optimal combination of channels.

**Table 5:** Average classification accuracies on DEAP achieved by SVM using all channels and partial channels selected by TS-MOEA.

**SVM using channels selected by TS-MOEA**		**SVM using all channels**
**Number of selected channels**	**Accuracy rate**	
30	72.08%	73.18%
27	69.38%	
22	71.88%	
17	71.63%	
12	71.25%	
7	66.09%	
2	60.01%	

## 5 Discussion

### 5.1 Investigate of the zero assignment parameter *s*

As shown in Line 10 of Algorithm 1, *s* is modulates the difficulty level in deleting channels. Specifically, for the *k*^*th*^ sample, if the correlation coefficients between the *j*^*th*^ channel and more than *s* other channels are 0 in the filtered correlation matrix, then the *j*^*th*^ channel can be deleted. In this case, Setting *s* to a large value makes it challenging to meet the channel deletion criteria, potentially leading the algorithm into stagnation. Conversely, a small *s* might result in erroneous deletion of channels.

In this section, Subjects 1, 5, 6, 7, and 9 are chosen to analyze the impact of different *s* values on the effectiveness of the proposed TS-MOEA. *s* takes values within the range of [35, 55] with a step size of 5. The average HV values across 30 independent runs for different *s* on the selected five subjects can be found in Table 6. It can be observed that *s* = 40 achieves the highest average HV values. Taking the number of selected channels as 2, 12, 22, 32, 42, 52, and 60 as examples, Table 7 provides the average classification accuracies of TS-MOEA for different values of s. As shown in Table 7, when *s* = 40, the algorithm achieves the optimal classification accuracy for most of the lead selection schemes. Hence, in this paper, *s* takes the value of 40.

**Table 6:** Average HV values for different *s*.

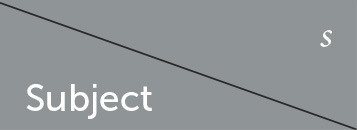	**35**	**40**	**45**	**50**	**55**
Subject 1	**6.62E+02**	**6.62E+02**	5.25E+02	4.05E+02	6.39E+02
Subject 5	6.02E+02	**6.56E+02**	6.02E+02	3.37E+02	3.94E+02
Subject 6	6.28E+02	**6.95E+02**	6.26E+02	5.05E+02	4.86E+02
Subject 7	5.70E+02	**6.39E+02**	5.94E+02	4.61E+02	5.28E+02
Subject 9	4.47E+02	4.45E+02	**4.82E+02**	3.27E+02	3.74E+02
Mean	5.82E+02	**6.14E+02**	5.66E+02	4.07E+02	3.69E+02

**Table 7:** Average classification accuracies of different *s* for all subjects.

**Number of selected channels**	**s**				
	**35**	**40**	**45**	**50**	**55**
60	98.40%	**99.20%**	98.14%	98.78%	99.18%
52	98.40%	**98.50%**	97.53%	97.56%	97.32%
42	96.70%	97.30%	95.61%	95.90%	**97.87%**
32	95.59%	**95.73%**	95.12%	93.85%	95.67%
22	91.92%	88.63%	84.88%	90.18%	**93.07%**
12	77.89%	**79.13%**	77.00%	77.10%	78.46%
2	77.27%	**78.13%**	77.00%	76.98%	77.23%

### 5.2 Investigate of the transition control parameter μ

It can be observed in Algorithm 2 that the parameter μ governs the transition from the early stage to the late stage in TS-MOEA. As described in Line 4 of Algorithm 2, the algorithm will shift from the early stage to the late stage if the number of function evaluations consumed by the early stage exceeds the predefined t, μ × *MaxFE*, or if the obtained Pareto-optimal solutions satisfy the diversity requirement. Therefore, if μ is set too large, the algorithm may exhaust a significant number of function evaluations in the early stage, potentially leading to unnecessary computational waste. Conversely, If μ is too small, the early stage might fail to produce solutions with a good distribution. Consequently, the late stage may not achieve satisfactory optimization results, given that the solutions obtained in the early stage serve as initial solutions in the late stage.

In this section, the influence of different μ on TS-MOEA's performance is investigated using five selected subjects: Subjects 1, 5, 6, 7, and 9. μ is within the interval [0, 1] using an increment of 0.2. When μ = 0, TS-MOEA exclusively executes the early stage, while μ = 1 means that only the late stage is executed. Table 8 provides the average HV values obtained from 30 independent runs for different μ values across the selected five volunteers. Statistical results indicate that the performance of TS-MOEA that operates in only one stage (μ = 0 and μ = 1) is inferior to that of the algorithm utilizing both stages concurrently (μ = 1/5, μ = 2/5, μ = 3/5, and μ = 4/5). As shown in Table 8, μ = 1/5 achieves the best results for 2 out of 5 subjects. However, μ = 1/5 also obtains the optimal mean values across all selected subjects. Table 9 gives the average classification accuracies of different μ for all subjects and μ = 1/5 achieves the best performance for most cases. Therefore, μ is set to 1/5 in this paper.

**Table 8:** Average HV values for different μ.

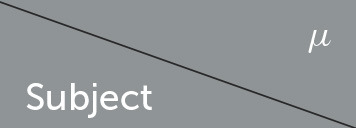	**0**	**1/5**	**2/5**	**3/5**	**4/5**	**1**
Subject 1	**7.85E+02**	6.21E+02	5.95E+02	6.34E+02	5.87E+02	2.12E+01
Subject 5	1.61E+01	**6.57E+02**	6.22E+02	6.40E+02	5.82E+02	1.51E+01
Subject 6	1.64E+01	5.30E+02	**5.69E+02**	5.39E+02	5.24E+02	1.88E+01
Subject 7	3.70E+02	5.49E+02	5.60E+02	**5.84E+02**	5.40E+02	1.69E+01
Subject 9	1.22E+01	**5.61E+02**	4.20E+02	4.77E+02	4.38E+02	1.18E+01
Mean	2.40E+02	**5.83E+02**	5.53E+02	5.75E+02	5.34E+02	1.68E+01

**Table 9:** Average classification accuracies of different μ for all subjects.

**Number of selected channels**	μ					
	**0**	**1/5**	**2/5**	**3/5**	**4/5**	**1**
60	91.98%	**92.75%**	91.66%	92.32%	90.98%	90.66%
52	90.82%	90.47%	90.50%	**90.86%**	89.20%	90.50%
42	**89.28%**	89.14%	88.25%	88.92%	87.97%	88.25%
32	86.57%	**88.57%**	87.62%	86.94%	86.05%	87.42%
22	85.46%	**86.14%**	85.55%	86.57%	85.42%	85.55%
12	83.23%	**84.36%**	83.47%	83.71%	84.02%	83.47%
2	70.15%	**76.85%**	75.59%	76.29%	74.47%	75.59%

### 5.3 Investigation of the distance radius *R*

In TS-MOEA, the distance radius *R* is adopted to calculate the scores of decision variables as depicted in Equation (9). If the distance between two channels is less than *R*, the score value assigned to these channels will be small, leading to a corresponding small value in the decision variable. Consequently, the correlation coefficient between the aforementioned channels is less likely to become 0 after filtering (Algorithm 4). If *R* is set to a small value, the probability of considering two channels as unrelated becomes low. In this case, the algorithm might retain channels that are useless to the specific task.

**Figure 11:**
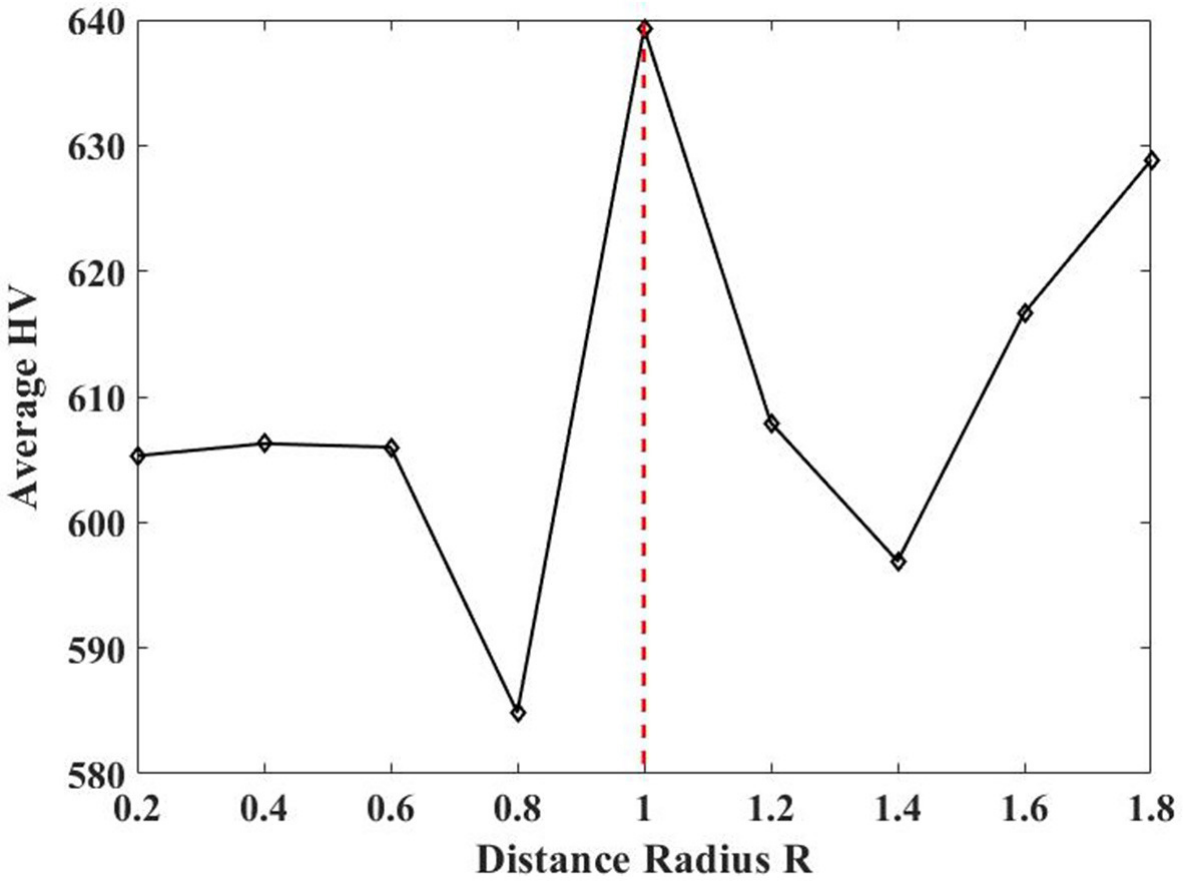
Average HV values of different *R*.

In this study, the distance radius *R* varies from 0.2 to 2 with increments of 0.2, as the maximum distance between two channels is 2. Figure 11 displays the average HV values obtained by TS-MOEA with different *R* across all subjects by 30 independent runs. It is evident from Figure 11 that the best performance is achieved when *R* = 1.0. Table 10 gives the average classification accuracies of different R for all subjects and *R* = 1.0 achieves the best performance for most cases. Therefore, the distance radius *R* is set to 1.0 in this paper.

**Table 10:** Average classification accuracies of different R for all subjects.

**Number of selected channels**	***R***								
	**0.2**	**0.4**	**0.6**	**0.8**	**1**	**1.2**	**1.4**	**1.6**	**1.8**
60	91.57%	92.55%	91.79%	92.18%	**92.58%**	91.35%	92.07%	92.79%	92.05%
52	90.13%	90.85%	90.86%	90.85%	**91.82%**	90.91%	91.61%	91.40%	91.13%
42	89.57%	90.05%	89.75%	89.94%	**90.63%**	89.37%	90.36%	90.42%	89.15%
32	87.94%	88.26%	**88.67%**	87.86%	88.50%	88.19%	87.67%	88.17%	86.70%
22	**86.40%**	85.40%	84.95%	84.68%	86.04%	84.63%	84.20%	84.63%	84.90%
12	**82.52%**	80.66%	82.08%	79.98%	82.36%	80.97%	81.17%	81.13%	79.64%
2	71.80%	63.43%	73.88%	65.57%	**74.40%**	63.45%	63.40%	63.06%	65.93%

### 5.4 Investigation of the selected channels

**Figure 12:**
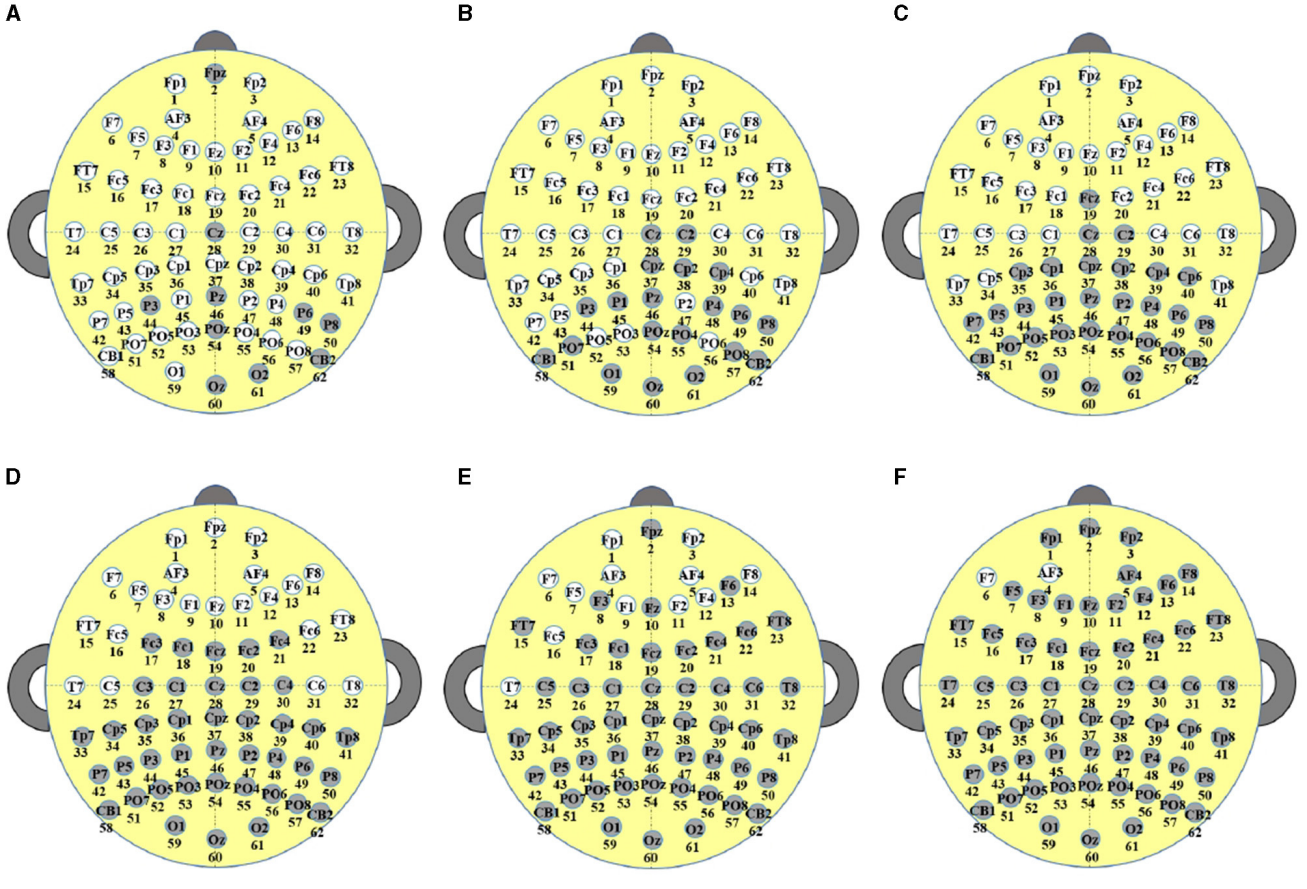
The selected channels for all subjects by TS-MOEA, **(A–F)** illustrate the scenarios with selected numbers of channels at 52, 42, 32, 22, 12, and 2, respectively (where the gray circles represent channels that have been deleted, and the white circles represent channels that have been selected).

This section discusses the channels selected by the proposed TS-MOEA, using the fatigue detection task in Section 4.2 as an example. Figure 12 illustrates the scenarios with selected numbers of channels at 52, 42, 32, 22, 12, and 2 for all subjects, and Table 3 shows the corresponding average classification accuracies for the six cases. As shown in Figure 12 and Table 3, the average classification accuracy gradually decreases as the number of deleted channels increases. It can be observed that the channels selected from Figures 12A–D essentially include the frontal (Fpz, F3, Fz, F6), frontotemporal (FT7, Fc6, FT8), and central (C5, C6, T8) regions. Specific activity patterns in the frontotemporal region may be associated with dreams and cognitive activity during sleep. Activity in the central regions may be associated with motor inhibition and somatosensory information processing during sleep, and activity patterns in these regions may reflect changes in muscle relaxation and sensory information transfer during sleep. From Figures 12D–F, the aforementioned ten channels were deleted, and there is a noticeable decline in classification accuracy, which can be seen in Table 3. Therefore, the removal of channels from regions closely related to the fatigue detection task will result in a sharp decline in classification accuracy. From this, it can be understood that incorporating prior knowledge of regions of interest (ROIs) related to specific tasks into the lead selection algorithm may be beneficial. For instance, in the context of fatigue detection tasks as discussed in Section 4.2, prioritizing the retention of channels from the frontal, frontotemporal, and central regions can help the channel selection algorithm strike a balance between the number of leads chosen and the classification accuracy.

## 6 Conclusions

This paper introduces a two-stage sparse multi-objective evolutionary algorithm (TS-MOEA) to solve channel selection problems within BCIs. In TS-MOEA, a two-stage framework with two different two-objective problem models has been adopted. Specifically, a two-objective problem which is sensitive to channel deletion is used in the early stage of TS-MOEA to prevent the algorithm from stalling. In the late stage of TS-MOEA, a two-objective problem model that can directly indicate the number of deleted channels is utilized. To strike a balance between convergence and population diversity in TS-MOEA, a transition condition has been devised. This condition takes into account both the number of consumed function evaluations and the distribution of the current population to control the transition between the early and late stages of the proposed algorithm. Moreover, due to the sparsity of the correlation matrix of channels, a sparse initialization operator is introduced to generate the initial population. Furthermore, a *Score*-based mutation operator has been integrated to enhance the search efficiency of the early stage in TS-MOEA. The experimental results of TS-MOEA and five other advanced MOEAs have demonstrated the efficiency of the proposed algorithm. However, as shown in Figure 8, Tables 3, 5, TS-MOEA provides a set of Pareto-optimal solutions, each offering a different channel selection scheme. Therefore, TS-MOEA does not directly yield a single optimal electrode selection scheme; in practical applications, the user must make a decision on which channel selection scheme to choose from the Pareto-optimal solution set. Additionally, although TS-MOEA takes into account knowledge relevant to the problem, such as channel positions and the distance matrix between channels, it does not consider the impact of regions of interest (ROI) on the performance of the algorithm.

As shown in Section 3.2, TS-MOEA incorporates the problem-domain knowledge, specifically the locations and distance matrix of channels, to enhance the algorithm's performance. However, the biological connections between brain regions, which could better capture and exploit correlations between different channels, were not considered. Therefore, how to combine the biological connections between brain regions in the design of critical operators to improve the search capabilities of the algorithm is one of the future works of this paper. Furthermore, as the number of commands increases, the number of brain wave patterns (or other physiological signals) that the BCI system needs to distinguish becomes larger, which increases the complexity of the classification task. Therefore, how to maintain or improve classification accuracy when the number of commands increases will be one of the future works of this paper.

## Data availability statement

The data analyzed in this study is subject to the following licenses/restrictions: the data that support the findings of this study are available on request from the corresponding author upon reasonable request. Requests to access these datasets should be directed to liuty@shmtu.edu.cn.

## Ethics statement

The studies involving human participants were reviewed and approved by the Medical and Life Science Ethics Committee of Tongji University. Written informed consent to participate in this study was provided by the participants.

## Author contributions

TL: Conceptualization, Formal analysis, Funding acquisition, Investigation, Methodology, Supervision, Validation, Writing—original draft, Writing—review & editing. YW: Data curation, Methodology, Formal analysis, Software, Visualization, Writing—review & editing. AY: Conceptualization, Data curation, Methodology, Software, Visualization, Writing—original draft, Writing—review & editing. LC: Data curation, Funding acquisition, Validation, Writing—original draft. YC: Data curation, Writing—review & editing.
